# Human periostin gene expression in normal tissues, tumors and melanoma: evidences for periostin production by both stromal and melanoma cells

**DOI:** 10.1186/1476-4598-6-80

**Published:** 2007-12-17

**Authors:** Gaëlle Tilman, Marina Mattiussi, Francis Brasseur, Nicolas van Baren, Anabelle Decottignies

**Affiliations:** 1 Cellular Genetics Unit, de Duve Institute, Catholic University of Louvain, B-1200 Brussels, Belgium; 2 Ludwig Institute for Cancer Research, Brussels Branch, B-1200 Brussels, Belgium

## Abstract

**Background:**

Recently, periostin (*POSTN*), a gene encoding a protein with similarity to the fasciclin family and involved in cell survival and angiogenesis, has emerged as a promising marker for tumor progression in various types of human cancers. There is some controversy regarding both *POSTN *expression levels and the nature of periostin-producing cells within tumors. In this study, we used quantitative RT-PCR to assess periostin gene expression in normal tissues, primary cell cultures, tumor tissues and tumor cell lines.

**Results:**

Periostin expression levels are highly variable in both normal tissues and tumors and strong *POSTN *overexpression is mostly detected in tumors from pancreas and liver. *POSTN *is not expressed in blood cancers. In melanoma samples, average periostin expression is not increased in primary tumors whereas *POSTN *overexpression was detected in about 60% of melanoma metastatic tumors in the liver or lymph nodes. Identification of the cellular source of periostin production in melanoma metastases -cancer cells or stroma- was assessed by comparing periostin expression in 23 newly-established melanoma cell lines and matched tumors. In contrast to the reduction by more than 99% of *COL6A3 *stromal marker mRNA in all cell lines, significant *POSTN *transcription was maintained in some melanoma cell lines, suggesting that both stromal cells and melanoma cells express periostin. The high level of periostin expression in primary cultures of skin fibroblasts suggests that fibroblasts may contribute for a large part to periostin production in melanoma-associated stroma. On the other hand, periostin expression in melanoma cells is probably acquired during the tumorigenic process as 1) normal melanocytes do not express *POSTN *and 2) melanoma cells from distinct metastases of the same patient were associated with very different levels of periostin expression.

**Conclusion:**

Our comparative analysis suggests that, although periostin overexpression is clearly detected in some cancers, it is not a general feature of tumors. In melanoma, our study identifies both stromal and melanoma cells as sources of periostin production and correlates *POSTN *expression levels with increased primary tumor thickness and metastatic process development.

## Background

A better understanding of the molecular mechanisms involved in melanoma and cancer progression in general is undoubtedly a major challenge in the development of new diagnostic and therapeutic approaches, underlying the necessity to identify new molecular targets. During the past decade, global gene expression profiling studies on various human cancer types, mainly relying on cDNA microarray technology, led to the identification of new candidate genes involved in cancer progression. These included periostin (*POSTN*), a gene encoding a secreted 90 kDa protein initially identified in a mouse osteoblastic library as a putative bone adhesion protein [1]. This protein shows sequence similarity to fasciclin I, an insect cell adhesion protein involved in central nervous system development [2], and human β IgH3, a TGF-β 1-induced protein promoting adhesion and spreading of dermal fibroblasts [3]. Binding of periostin to α_V_β_3 _and α_V_β_5 _integrins has been reported to promote cell adhesion and spreading and to activate the Akt/PKB signaling pathway leading to increased cellular survival and angiogenesis [4-6]. In pancreatic cancer cells, periostin was shown to bind to α_6_β_4 _integrin, thereby promoting phosphorylation of focal adhesion kinase and PKB through activation of the PI3 kinase pathway [7].

Over the past seven years, periostin was proposed to be a novel therapeutic target for cancer [8]. Indeed, *POSTN *gene was found to be overexpressed in various human cancers such as ovary [4,9], colon [6], pancreas [7,10], thyroid [11], oral squamous cell carcinoma [12,13], breast [5], lung [14] and neuroblastoma [15] and higher *POSTN *expression levels were correlated with increased tumor aggressiveness and/or poorer survival in NSLC [14,16], SCLC [17], neuroblastoma [15], colon cancers [6], thyroid carcinomas [11], oral squamous cell carcinoma [12] and pancreatic ductal adenocarcinoma [10]. However, other studies reported a down-regulation of *POSTN *transcription in bladder [18] and lung [19] cancer. The functional role of periostin in cancer is also under debate as both tumor-promoting [5-7,10,20,21] and tumor-suppressing activities [18,19] have been reported: on one hand, periostin was reported to increase invasiveness of tumor cell lines *in vitro *[7,12,20] but, on the other hand, periostin expression reduced invasiveness of bladder cancer cells [18] and decreased anchorage-independent growth of T24 bladder cancer cells and SaOS-2 osteosarcoma cell line [19]. *In vivo*, two reports demonstrated that *POSTN *overexpression in tumor cell lines increases metastasis and angiogenesis in nude mice and reduces stress-induced apoptosis [5,6] while another report provided evidence that periostin suppresses lung metastasis of mouse melanoma cell line B16-F10 [18].

Although *POSTN *overexpression does not seem to be systematic in human tumors, studies agree on the low level of periostin expression in most tumor cell lines [4,7,9,13,18,19,21]. Lower levels of *POSTN *expression in tumor cell lines compared to tumor tissues are in agreement with studies showing a production of periostin by stromal cells -and not cancer cells- in tumors [10,16,17,22]. However, the nature of periostin-producing cells in tumors is another matter of controversy as separate *in situ *hybridization experiments suggested that *POSTN *mRNA is detected in the cytoplasm of cancer cells [5,7].

In this study, we relied on quantitative RT-PCR to investigate *POSTN *expression in a series of human normal tissues and tumors. We next focused on cutaneous melanoma to quantify periostin transcripts in a total of 113 tumor samples, including primary and metastatic lesions, and we correlated periostin expression with Breslow thickness of melanoma primary tumors. Finally, to investigate the source of periostin production in melanoma, we analyzed *POSTN *transcription level in 23 newly-established melanoma cell lines and matched tumors and compared the results with the expression level of *COL6A3*, a melanoma-associated stromal marker encoding the α3 chain of collagen VI.

## Results

### *POSTN *expression in normal tissues

**Figure 1:**
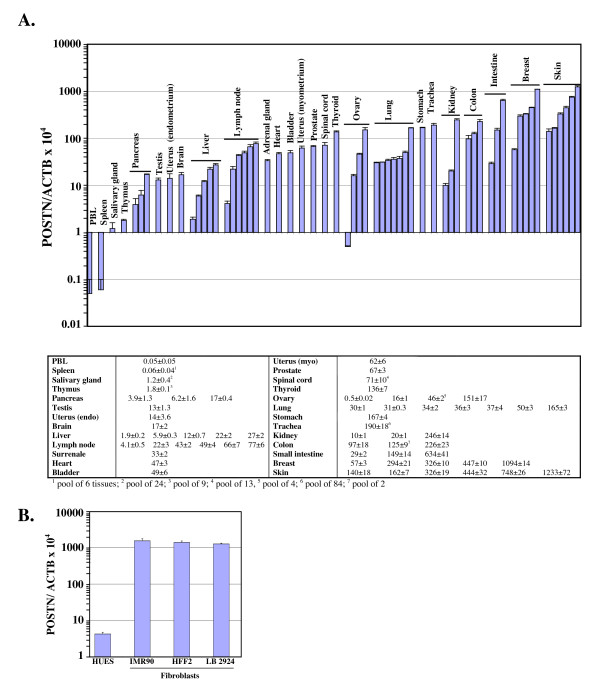
**Quantitative measurement of *POSTN *expression in human normal tissues and primary cultures**. *POSTN *and *ACTB *cDNA levels were measured by qRT-PCR based on Taqman technology. For each sample, measurements were done in triplicate. POSTN/ACTB × 10^4 ^was calculated as [(cDNA *POSTN*/cDNA *ACTB*) × 10^4^]. A. *POSTN *expression in normal tissues. Error bars represent standard deviations (SD). The table below the graph gives the average POSTN/ACTB × 10^4 ^± SD values for each sample. B. *POSTN *expression in primary cultures of human embryonic stem cells at day 5 (HUES), fetal lung fibroblasts (IMR90), newborn foreskin fibroblasts (HFF2) and adult skin fibroblasts (LB2924). Error bars represent SD.

We used qRT-PCR to quantify periostin expression in various normal human tissues. Values were normalized with the level of β-actin expression (Fig. 1A) and given as [(cDNA *POSTN*/cDNA *ACTB*) × 10^4^] (POSTN/ACTB × 10^4^). *POSTN *was expressed in a wide range of normal tissues but the expression was negligible in PBLs and spleen. Expression was also very low in salivary gland and thymus. Tissue samples from skin and breast were characterized by high *POSTN *expression (average expression of 509 ± 418 and 444 ± 390 respectively) although levels were fluctuating significantly between independent samples. Similarly, *POSTN *expression levels measured in four independent ovarian tissues spanned over a broad range (from 0.5 ± 0.02 to 151 ± 17). Conversely, periostin expression levels in normal pancreas, liver, lymph node, lung or colon were more homogeneous.

The above data suggest that tissues with low fibroblast content including PBLs, spleen, pancreas or liver show reduced *POSTN *expression compared to fibroblast-rich tissues like skin or breast. Together with previous observations that fibroblasts may be the source of periostin expression in various tumors [10,14,16,17], this suggests that fibroblast content may modulate *POSTN *expression level in normal tissues. To evaluate *POSTN *expression in fibroblasts, we extracted RNA from fibroblast primary cultures derived from either fetal lung (IMR90), newborn foreskin (HFF2) or adult abdominal skin (LB2924). *POSTN *expression levels as high as, respectively, 1547 ± 213, 1391 ± 142 and 1267 ± 57 were measured in these samples (Fig. 1B). This high level of *POSTN *expression in fibroblasts was not a consequence of establishing primary cultures as embryonic stem cell primary cultures (HUES) were characterized by very low periostin expression level (4.2 ± 0.5), further suggesting that periostin expression is not required at the blastocyst embryonic stage.

### *POSTN *expression in various tumors

**Figure 2:**
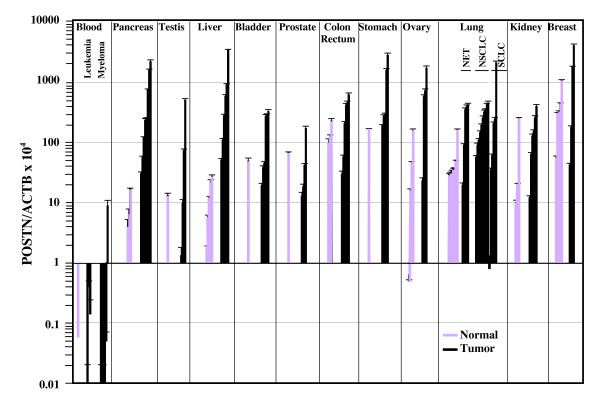
**Comparison of *POSTN *expression in normal tissues and tumors**. *POSTN *and *ACTB *cDNA levels were measured in triplicate in various normal tissues and tumors as described in Fig. 1 legend. Normal tissue samples correspond to samples from Fig. 1. POSTN/ACTB × 10^4 ^values are given as Mean ± SD in normal tissues (grey bars) and tumors (black bars).

To evaluate the extent of periostin expression in tumors, we measured the amount of *POSTN *and *ACTB *cDNA molecules in hematological malignancies and tumors from pancreas, testis, liver, bladder, prostate, colon, stomach, ovary, lung, kidney and breast (Fig. 2).

In line with the absence of *POSTN *expression in normal PBLs, periostin expression levels were negligible in hematological malignancies including leukemia (0.2 ± 0.2) and myeloma (1.5 ± 3.6). Overall, our data reveal the huge variability of *POSTN *expression levels in most solid tumors and normal tissues. Hence, increased periostin expression was only nearly significant in pancreatic adenocarcinoma (656 ± 819 *vs *9.1 ± 6.9 for normal pancreas, *P *= 0.06). In liver cancer, average *POSTN *expression was also increased (902 ± 1308, *n *= 6) compared to normal tissue samples (14 ± 11, *n *= 5) (*P *= 0.16). In ovarian tumors, *POSTN *expression levels were about 15-fold higher than the average level measured in a total of seven (three separate ovaries and one pool of four) normal tissues (759 ± 708 *vs *52 ± 66) (*P *= 0.14). For lung cancer, we measured a significant 5-fold increase of *POSTN *expression in NSCLC tumors (272 ± 177 *vs *55 ± 49 for normal lung, *P *= 0.002).

### *POSTN *expression in primary and metastatic melanoma lesions

**Figure 3:**
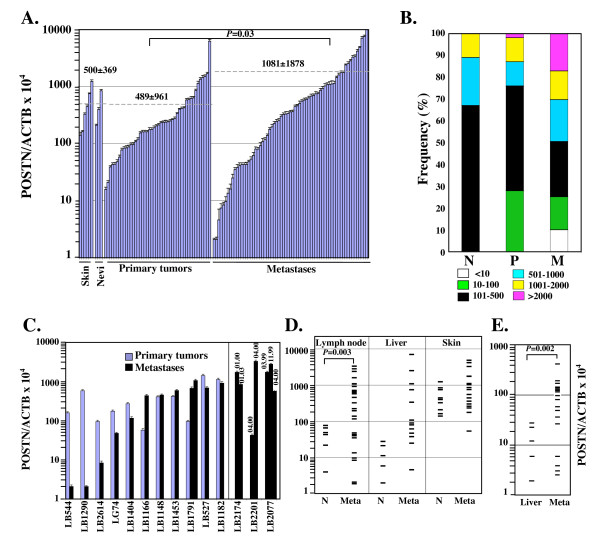
**Quantitative analysis of *POSTN *expression in melanoma**. A. *POSTN *and *ACTB *cDNA levels were measured in triplicate in normal skin (*n *= 6), benign nevi (*n *= 3) and melanoma primary tumors (*n *= 46) or metastases (*n *= 67) as described in Fig. 1 legend. Error bars represent SD. B. Data from panel A for normal tissues (N), primary tumors (P) and metastases (M) were classified into six ranges of POSTN/ACTB × 10^4 ^values. C. Comparison of POSTN/ACTB × 10^4 ^values between metastases and matched primary tumors from 11 patients or between distinct metastases from the same individual (patients LB2174, LB2201 and LB2077, with indication of tumor resection dates as MM.YY). Error bars represent SD. D. Metastatic tumors (Meta) were classified according to their localization (lymph node, liver or (sub)-cutaneous) and *POSTN *expression levels were compared to levels in tissue samples from normal organs (N). E. POSTN/ACTB × 10^4 ^values were measured in hepatic metastatic lesions from intraocular melanoma (Meta) and normal liver.

Next, we investigated *POSTN *expression in 113 human cutaneous melanoma samples, including 46 primary lesions and 67 metastases. Periostin expression in melanoma was compared to the expression level in nine normal skin or benign nevus tissues (Fig. 3A). The average *POSTN *expression levels amounted to 500 ± 369 in normal tissues, 489 ± 961 in primary tumors and 1081 ± 1878 in metastases. Although periostin expression levels were variable in melanoma tumors (Fig. 3A and 3B), average *POSTN *expression level was significantly higher in metastases compared to primary lesions (*P *= 0.03).

Melanoma metastases were characterized by highly variable *POSTN *expression levels. Notably, very low expression levels (<10) were measured in 9% of melanoma metastases while 15% of them showed very high levels of *POSTN *expression (>2000) (Fig. 3B). To further compare periostin expression in primary tumors and metastases, we measured the level of *POSTN *transcripts in metastases and matched primary tumors from 11 patients (Fig. 3C). No correlation was found: *POSTN *expression was either reduced (by up to 280-fold), maintained or increased (by up to 11-fold) in metastases compared to primary melanoma. Further analysis also revealed that *POSTN *transcription levels may vary significantly between distinct metastases isolated from the same individual (patients LB2174, LB2201 and LB2077, Fig. 3C). These variations may be explained by distinct localization of melanoma metastases as normal skin produces significant amounts of periostin unlike other organs with very low endogenous levels of periostin, like lymph nodes or the liver. Strikingly, our analysis revealed a significant increase of *POSTN *expression in more than 60% of tumor-invaded lymph nodes (*P *= 0.003) (Fig. 3D). Conversely, tumor-invaded lymph nodes with low *POSTN *expression account for the low levels (<100) measured in a subset of melanoma metastases. Similar observations were made with hepatic melanoma metastases (Fig. 3D). Hence, these data demonstrate the *POSTN *overexpression in about 60% of melanoma metastases in the liver or lymph nodes. Periostin overexpression in (sub)cutaneous melanoma metastases is more difficult to assess as *POSTN *transcripts are already abundant in normal skin (Fig. 3D). *POSTN *overexpression in metastatic tumors was confirmed by an analysis of 19 hepatic metastases from intraocular melanoma (Fig. 3E) (*P *= 0.002).

**Figure 4:**
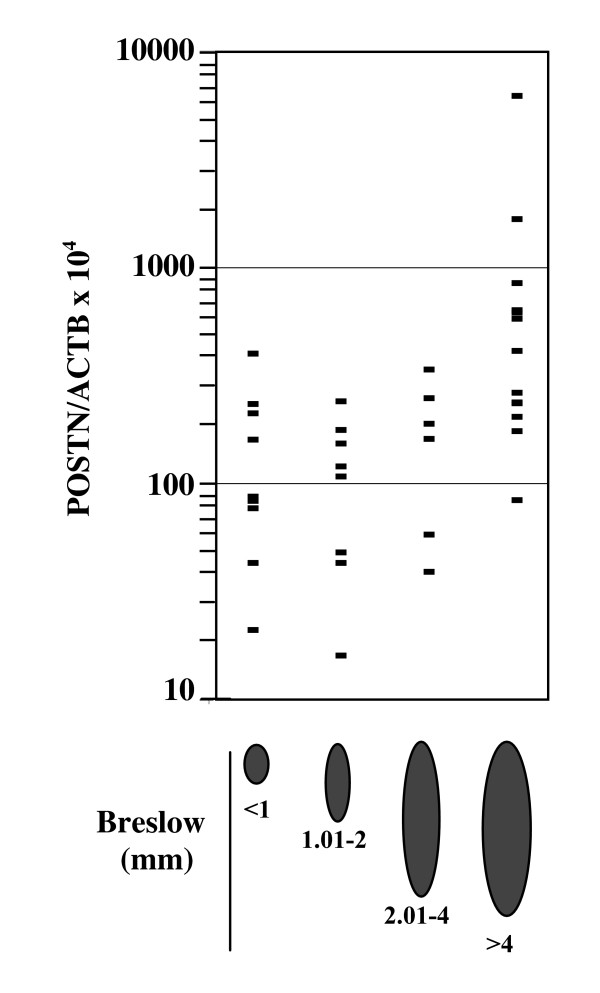
**Correlation between *POSTN *expression and Breslow thickness of melanoma**. *POSTN *and *ACTB *cDNA levels were measured in triplicate in primary tumors of melanoma with Breslow thickness <1.0 mm (*n *= 9), 1.01<>2.0 mm (*n *= 8), 2.01<>4.0 mm (*n *= 6) and >4.0 mm (*n *= 12) as described in Fig. 1 legend.

Earlier reports suggested that periostin expression level was correlated with tumor aggressiveness and/or poorer survival in NSCLC [14,16], SCLC [17], neuroblastoma [15], colon cancers [6], thyroid carcinomas [11], oral squamous cell carcinoma [12] and pancreatic cancer [10]. Here, we relied on a revised version of Breslow classification based on primary tumor thickness [23] to correlate periostin expression with prognosis of melanoma (Fig. 4). Quantitative measurement of *POSTN *expression was performed on a total of 35 primary melanoma lesions. *POSTN *expression levels >300 were measured in only 9% of tumors with Breslow thickness ≤ 4 mm but were detected in 58% of tumors with Breslow thickness >4 mm. These data suggest that, in melanoma, increased tumor thickness may be correlated with increased periostin expression although tumoral cell content of samples is difficult to estimate and may vary with tumor thickness.

### *POSTN *expression in melanoma cell lines

To gain more insight into the nature of periostin-producing cells in metastatic lesions, we measured *POSTN *expression levels in newly established melanoma cell lines and matched tumors. One major difference between melanoma tumor and cell line cDNA samples is the presence of stromal cell cDNA in tumors whereas cell lines derive from malignant cells exclusively.

**Figure 5:**
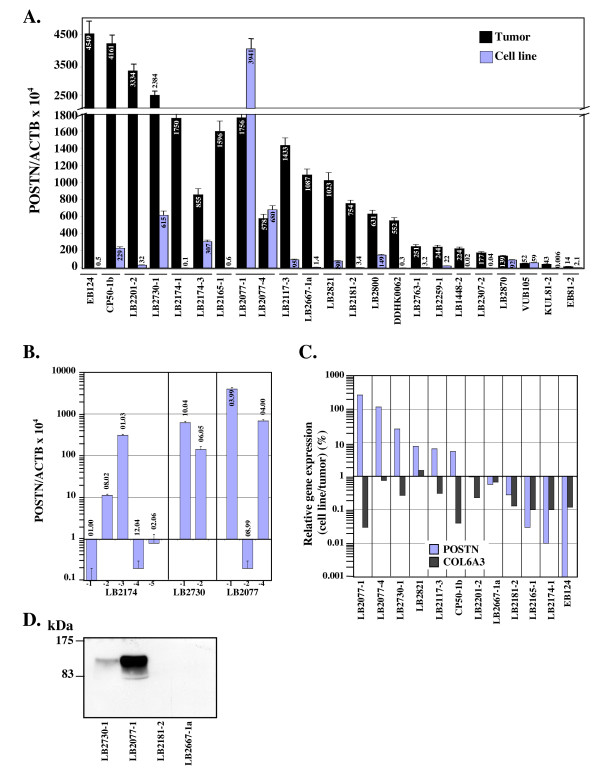
**Comparison of *POSTN *expression in melanoma tumors and cell lines**. A. A total of 23 melanoma cell lines (black bars) and matched metastatic tumors (grey bars) were assessed for *POSTN *and *ACTB *expression in triplicate as described in Fig. 1 legend. All melanoma cell lines were established in our laboratory and subjected to no more than 4 to 11 passages before RNA extraction. Metastases were isolated from tumor-invaded lymph nodes (LB2201-2, LB2730-1, LB2165-1, LB2117-3, LB2667-1a, LB2800, LB1448-2, LB2870, EB81-2), skin (DDHK0062, KUL81-2), sub-cutaneous tissues (EB124, CP50-1b, LB2174-1, LB2174-3, LB2077-1, LB2821, LB2181-2, LB2763-1, LB2259-1, LB2307-2, VUB105) or heart (LB2077-4). Error bars represent SD. B. *POSTN *expression level was measured in independent melanoma cell lines derived from distinct metastasis of patients LB2174, LB2730 and LB2077. Date of cell line establishment (MM.YY) is indicated above the bars. C. *POSTN *and *COL6A3 *cDNA levels were measured by qRT-PCR in 12 melanoma cell lines and matched tumors. Values are given as (expression in cell line/expression in tumor) × 100%. D. Periostin protein was analyzed in conditioned medium from LB2077-1 and LB2730-1 periostin-producing melanoma cell lines and from LB2181-2 and LB2667-1a melanoma cell lines, which do not express *POSTN*, as negative controls.

We measured the level of *POSTN *transcripts in 23 melanoma cell lines and matched tumors (Fig. 5A). These melanoma lines were established in our laboratory and were subjected to no more than 4 to 11 passages before *POSTN *expression analysis. Overall, *POSTN *transcript abundance was decreased in most cell lines compared to tumors and expression levels were extremely low (from 0.06 ± 0.01 to 3.4 ± 0.3) in 11 cell lines. However, six cell lines, LB2077-1, LB2077-4, LB2730-1, LB2174-3, CP50-1B and LB2800, were characterized by higher periostin expression (between 149 ± 21 and 3941 ± 362) and, as expected for a secreted protein, periostin was detected in the conditioned medium (Fig. 5D). Interestingly, independent cell lines derived from distinct metastases of the same patient were associated with very different levels of periostin expression (LB2077 and LB2174 patients, Fig. 5A and 5B). Notably, *POSTN *expression level was <1 in LB2077-2 cell line derived from a breast metastasis whereas periostin was expressed at very high level in LB2077-1 (3941 ± 362) and LB2077-4 (680 ± 73) cell lines derived from metastases located, respectively, in scalp sub-cutaneous tissues and heart of the same patient. Data also suggest that high level of periostin expression in metastatic tumoral cells is not time-dependent as variable expression levels were observed during the tumor resection history of melanoma patients LB2174 and LB2077 (Fig. 5B).

To follow the disappearance of stromal fibroblasts upon establishment of melanoma cell lines from tumor samples, we searched for a genetic marker of fibroblasts in melanoma. cDNA microarray analysis of a series of paired melanoma tumor and tumor-derived cell line samples revealed that a set of stromal genes, including the gene encoding the α3 chain of collagen VI, *COL6A3*, were systematically and significantly down-regulated in all cell lines (data not shown). *COL6A3 *encodes a fibrillar protein of the extracellular matrix believed to be involved in cell anchoring and signaling through interactions with integrins [24] and induced by TGF-β1 in dermal fibroblasts [25], suggesting that *COL6A3 *is a good genetic marker for melanoma-associated stromal fibroblasts. Hence, we compared *COL6A3 *expression levels in 12 melanoma cell lines and matched tumors by qRT-PCR (Fig. 5C). In agreement with microarray data, *COL6A3 *expression was drastically reduced in all cell lines tested compared to matched tumors with cell line/tumor ratio of *COL6A3 *cDNA ranging from 0.03 to 1.5% (Fig. 5C). By comparison, cell line/tumor ratio of *POSTN *expression was smaller than 1% in only six cases; the ratio was comprised between 5 and 8% in three cases and higher than 25% in LB2077-1, LB2077-4 and LB2730-1.

**Figure 6:**
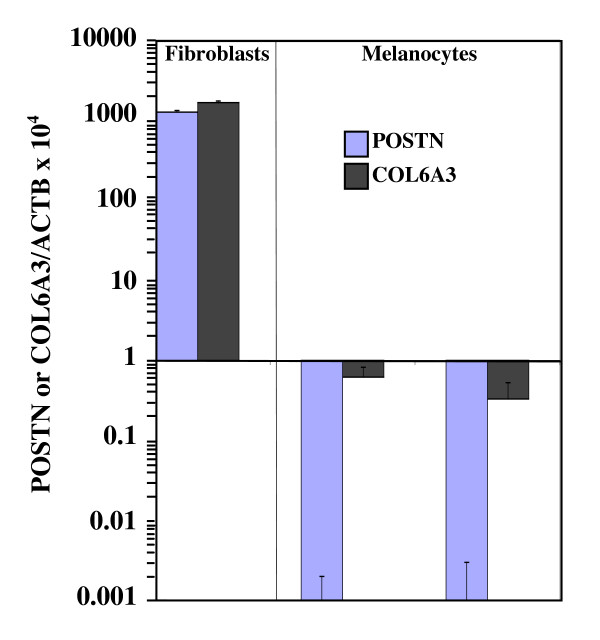
**Quantitative measurement of *POSTN *and *COL6A3 *expression in primary cultures of skin fibroblastes and melanocytes**. *POSTN*, *COL6A3 *and *ACTB *cDNA levels were measured in triplicate in primary cultures of skin fibroblasts (LB2924) and melanocytes (NHEM and LB656). COL6A3/ACTB × 10^4 ^was calculated as [(cDNA *COL6A3*/cDNA *ACTB*) × 10^4^]. Error bars represent SD.

All together, these data suggest that, in about half of the melanoma samples tested, *POSTN *expression is restricted to stromal cells while, in the other half, cancer cells are another source of periostinproduction. The detection of *POSTN *expression in a fraction of melanoma cell lines suggests either that normal melanocytes express periostin and that the expression is maintained in some melanoma cells or that normal melanocytes do not express periostin but may acquire the ability to express the gene during the tumorigenic process. To address that issue, we quantified periostin expression in normal human melanocytes. Unlike the expression profile detected in skin fibroblasts, *POSTN *expression was not detected in melanocytes and *COL6A3 *expression was very low (Fig. 6). Therefore, our data suggest that, in metastatic melanoma tumors, periostin expression can be induced in melanoma cells.

### *POSTN *expression in other cancer cell lines

Finally, to compare the periostin expression levels in other cancer cell lines with the values obtained in melanoma, we selected a series of 19 cell lines derived from tumors of various cancer types (Table 1). *POSTN *expression levels <5 were detected in 16/19 cancer cell lines whereas strong expression was detected in Hs578T breast cancer cell line and LB831 bladder carcinoma cell line. Intermediate expression level of 45 ± 4 was measured in A172 glioblastoma cell line. Hence, these data suggest that expression of periostin by cancer cells is not restricted to melanoma cells but may also occur in other cancer types.

**Table 1:** Periostin expression in various cancer cell lines

**Cell line**	**Origin**	**POSTN/ACTB^1^**
U2OS	Osteosarcoma	3.5 ± 1.7
LB96	Ewing sarcoma	0
LB23-1	Rhabdomyosarcoma	0.1 ± 0.1
Hela	Cervical cancer	3.0 ± 0.4
PA-1	Ovarian teratocarcinoma	1.4 ± 0.1
LB37-1	NSCLC	2.8 ± 0.6
LB85	SCLC	3.4 ± 0.2
LB92	SCLC	0.6 ± 0.2
LB1047	Renal cell carcinoma	0.8 ± 0.2
BB64	Renal cell carcinoma	0.08 ± 0.01
LB108	Colorectal cancer	0
MCF7	Breast cancer	0
Hs578T	Breast cancer	3693 ± 86
Panc-1	Pancreatic carcinoma	0
Capan-1	Pancreatic carcinoma	0
Huh-7	Hepatocarcinoma	0.3 ± 0.07
LB831	Bladder carcinoma	1748 ± 74
MZGC3	Stomach cancer	0
A172	Glioblastoma	45 ± 4
MZ2	Melanoma	2.3 ± 0.7
LB39	Melanoma	0.5 ± 0.03
LB2586-7	Melanoma	3.4 ± 0.3
LB2201-3	Melanoma	4.2 ± 0.4
A375	Melanoma	4.7 ± 1.2

**^1 ^**(cDNA *POSTN*/cDNA *ACTB*) × 10^4^

## Discussion

The discrepancy between different studies regarding the expression level of *POSTN *in normal tissues and the recent observation that transcription of the gene is up-regulated in various tumors [8] prompted us to set up a quantitative RT-PCR assay to measure *POSTN *mRNA in human normal tissues and tumors.

In normal tissues, previous Northen blot experiments demonstrated that periostin is not expressed in PBLs, highly expressed in fetal tissues or serum but studies disagreed on the extent of *POSTN *expression in a series of other tissues [4,19,21]. Our data confirm the absence of *POSTN *transcription in both PBLs and spleen, in agreement with previous observations [4,19]. Salivary gland, thymus and embryonic stem cells were also characterized by very low expression levels. The highest *POSTN *cDNA levels were measured in colon, small intestine, breast and skin. However, in the three latter organs, as well as in kidney and ovaries, expression levels were highly fluctuating between tissue samples, providing a possible explanation for the discrepancies between previous studies. The diversity in cell type content among samples may possibly account for this variability. In that respect, skin samples contain different cell types including fibroblasts, keratinocytes and melanocytes for which we have measured *POSTN *expression levels of, respectively, about 1500, 50 (data not shown) and 0.

In tumors, leukemia and myeloma showed negligible levels of periostin expression whereas *POSTN *transcripts were detected in all solid tumors. We detected a 72-fold increase of *POSTN *transcription in pancreatic adenocarcinoma compared to normal tissues (*P *= 0.06), in line with previous studies reporting a 42- to more than 100-fold increase of *POSTN *mRNA level in pancreatic tumors [7,10]. In liver and ovarian tumors, average expression levels were increased by 65- and 15-fold respectively. The only evidences for increased periostin expression in liver cancer reported so far come from immunohistochemical analysis of tumor tissues [7]. In ovarian tumors, tumor-derived epithelial cells and ascites, previous semi-quantitative studies reported elevated levels of periostin mRNA and protein [4,9]. Previous studies reported *POSTN *up-regulation in about half of the NSCLC tumors [14] and a slight increase in periostin serum levels from NSCLC patients [16]. Higher periostin serum levels were also correlated with increasing T- or N-stage in SCLC patients [17] but, on the other hand, *in situ *mRNA hybridization suggested a down-regulation of *POSTN *gene transcription in SCLC tumors [19]. Our data suggest that *POSTN *transcription is increased by 5-fold in NSCLC tumors. No significant difference was measured for SCLC and NET tumors although more samples should be analyzed. Analysis of six colorectal tumors and three normal colon samples revealed only a 2-fold increase in *POSTN *expression (*P *= 0.16). The modest increase in *POSTN *transcription that we measured in colorectal tumor samples does not match previous data from a semi-quantitative study reporting considerable periostin overexpression in 25/29 pairs of matched normal colon tissue and colon tumor samples [6]. The reason for this is unclear but may be related to the fact that Bao *et al. *mainly focused on colon cancers with hepatic metastases. In breast and kidney tumors, the difference in periostin expression was not significant due to the highly variable *POSTN *expression levels measured in both normal and tumoral samples. The absence of significant increase in *POSTN *expression in breast tumors contrasts with the conclusions from a previous study in which expression in tumors was compared to the low level of *POSTN *mRNA in primary mammary epithelial cultures as reference for normal breast tissue [5]. Overall, for most tumor types tested in this study, our data revealed highly variable *POSTN *expression levels in both tumors and normal tissues, suggesting that larger numbers of samples should be tested to address periostin expression in tumors more significantly.

In normal skin, melanocytes do not express *POSTN *but fibroblasts express the gene at high level. Investigation of 46 primary cutaneous melanoma lesions did not reveal any significant difference in average *POSTN *expression compared to normal tissues although we found that, in primary melanoma, thicker tumors (> 4 mm) may be correlated with increased periostin expression (*P *= 0.07). However, melanoma cell content of tumor samples is difficult to estimate and may vary with tumor thickness, leading to possible distortion of the data. In metastatic melanoma lesions, *POSTN *expression ranged from very low to very high levels compared to normal skin. Classification of melanoma metastases showed that very low *POSTN *expression levels are found in metastases located in organs with low endogenous periostin expression, like the liver or lymph nodes. In these organs, low *POSTN *expression levels are expected if melanoma cells do not produce periostin. Conversely, periostin levels should be high if melanoma metastases overexpress *POSTN *either through acquisition of *POSTN *expression by melanoma cells or to increased periostin expression in tumor-associated stromal cells. In that respect, about 60% of melanoma metastatic tumors in the liver or lymph nodes showed a clear *POSTN *overexpression compared to normal organs.

The nature of periostin-producing cells in tumors is still under debate as separate studies reported a production of periostin by stromal cells [10,16,17,22] whereas other experiments suggested that *POSTN *mRNA is detected in cancer cells [5,7]. To investigate the nature of periostin-producing cells in melanoma, periostin expression was measured in newly established melanoma cell lines and matched tumors. In cell lines, expression of *COL6A3 *stromal cell marker was reduced to less than 1% of the value measured in matched tumors whereas periostin was expressed (at either low or high level) in about half of the melanoma cell lines. Given that normal melanocytes do not express periostin, this study suggests that melanoma cells sometimes acquire the ability to express the gene during tumorigenesis. Our data also indicate that cell lines isolated from distinct metastases of the same patient may be characterized by either negligible or high *POSTN *expression, suggesting that periostin expression is acquired by melanocytes during the metastatic process. Previous studies reported low *POSTN *expression in most tumor-derived cell lines, whatever their origin [4,7,9,13,18,19,21] but most cancer cell lines tested had been established a long time before and subjected to extensive culture, making it difficult to establish whether the decreased expression in cancer cell lines was reflecting a loss of stromal-associated *POSTN *expression as suggested by some authors [10,16,17,22] or artifacts due to prolonged cell culture. The induction of periostin expression in melanoma cells may be a consequence of mutagenic events occuring during the tumorigenic processes or may be the result of interactions with stromal components previously reported to influence development and progression of carcinomas [26]. In that respect, earlier experiments showed an induction of *POSTN *expression in tumors recovered from nude mice injected with periostin-negative transformed cell lines [21] (although one cannot rule out the possibility that stromal cells themselves may be the source of periostin expression in these tumors). On the other hand, our data indicate that stromal cells are an important source of periostin production in melanoma tumors. In some instances, periostin expression levels were higher than 3000 in the tumor but negligible in matching cell lines, suggesting that periostin expression may be very high in tumor-associated stromal cells. In line with this, a significant increase in periostin expression was measured in fibroblast-like stellate cells from pancreatic ductal adenocarcinoma tumors, a cancer characterized by excessive desmoplasia [10]. Stromal cells have a prominent role in cancer progression and cancer-associated fibroblasts are believed to play a crucial role by producing growth factors, chemokines and extracellular matrix components that promote tumor angiogenesis [27].

The increased expression of periostin in melanoma metastatic lesions that we identified in this study is in agreement with *in vivo *studies revealing that periostin overexpression promotes metastatic growth of cancer cells [5,6,20,28]. Hence, this work suggests that, in melanoma, periostin overexpression may also be involved in the process of metastasis. In line with this, two previous studies identified an up-regulation of integrin αV (ITGAV), one of the subunits of periostin receptor, as a predictive marker for melanoma metastasis in primary tumors [27,29,30].

## Conclusion

Periostin was reported to promote cell survival and angiogenesis in tumors. Although cancer-associated *POSTN *overexpression is well documented for some tumor types, it is still uncertain for other ones. To date, no quantitative comparison of periostin expression in a large panel of normal and tumor tissues was available. In this study, we detected considerable variability of *POSTN *transcript levels among samples from the same tissue type, especially for tissues with variable stroma content. Overall, our results support previously reported increase of periostin expression in tumors from pancreas, liver and NSCLC but suggest that *POSTN *overexpression is not a general feature of tumors. In melanoma, *POSTN *transcription was not increased in primary lesions compared to normal skin but was correlated with Breslow thickness. On the other hand, about 60% of melanoma metastases from lymph nodes or the liver overexpress *POSTN*. Analysis of newly-established melanoma cell lines and matched metastases revealed that, although stromal cells -presumably fibroblasts- are mostly responsible for periostin production in melanoma metastases, melanoma cells sometimes acquire the ability to express *POSTN *at high level. This acquisition is associated with the metastatic process as distinct metastases from the same patient were associated with very different *POSTN *expression levels. Hence, this work reconciles previously published studies reporting conflicting data about the nature of periostin-producing cells in tumors (stromal or cancer cells) and identifies *POSTN *as a marker of metastasis in melanoma.

## Methods

### Chemicals

Minimum Essential Medium (MEM), Iscove's Modified Dulbecco's Medium (IMDM), Dulbecco's Modified Eagle Medium (DMEM), trypsin-EDTA and essential amino acids were purchased from GIBCO (Invitrogen, Merelbeke, Belgium); fetal calf serum was from HyClone (Perbio Science, Aalst, Belgium); TriPure reagent from Roche Applied Science Diagnostics (Mannheim, Germany) and all other reagents were from Sigma Aldrich (Bornem, Belgium).

### Primary cultures, normal tissues, tumors and cell lines

IMR90 fetal lung fibroblasts were kindly provided by M. Ricchetti (Institut Pasteur, Paris, France); HFF-2 newborn foreskin fibroblasts were purchased from ATCC (Rockville, MD); LB2924 fibroblasts were isolated from adult abdominal skin in our laboratory and normal human epidermal melanocytes NHEM derived from foreskin were purchased from PromoCell GmbH (Heidelberg, Germany). Normal tissue and tumor samples were obtained from patients undergoing surgery or tumor resection between 1991 and 2006. Experimental procedures involving the use of biological material were approved by our Institutional Review Board. All patients gave informed consent. In general, tumor biopsies were obtained as part of screening procedures for participation in clinical immunotherapy trials. The informed consent mentioned that part of the tumor samples could be used for research purposes. In a few cases, anterior to 2002, oral informed consent was obtained. Surgical specimens were immediately frozen in liquid nitrogen and stored at -80°C until RNA extraction. Cell lines derived from melanoma, rhabdomyosarcoma, NSCLC, SCLC, renal cell carcinoma, bladder carcinoma and larynx epidermoid carcinoma were derived from patient specimens in our laboratory. HeLa, PA-1, A172, A375, Hs578T, MCF-7 and Capan-1 cell lines were purchased from ATCC. Cell lines derived from osteosarcoma (U2OS), hepatocarcinoma (Huh-7), stomach tumor (MZGC3) and pancreatic carcinoma (Panc-1) were kindly provided by, respectively, F. Fuks (Université Libre de Bruxelles, Brussels, Belgium), A.H. Patel (University of Oxford, Oxford, UK), P. Coulie (Université Catholique de Louvain, Brussels, Belgium) and C. Hill (Cancer Research UK, London, UK). Fibroblasts were maintained as a monolayer in MEM media supplemented with 1% essential amino acids at 37°C in a humidified atmosphere saturated with 5% CO_2_. All cancer cell lines were grown at 37°C in a humidified atmosphere saturated with 8% CO_2 _in IMDM except for U2OS and HeLa (DMEM) and MZ2 and LB23-1 (DMEM/Hepes/glucose) cell lines. All media were supplemented with 10% fetal calf serum, 100 U/ml penicillin and 100 μg/ml streptomycin.

### RNA extraction and reverse transcription

Total cellular RNA was extracted with TriPure reagent or by the guanidine-iosthiocyanate/cesium-chloride procedure [31]. RNA samples from 18 normal human tissues were purchased from either Clontech (Mountain View, CA) or AMBION (Austin, TX). RNA from LB656 melanocytes was kindly provided by L. Old (Ludwig Institute for Cancer Research, New-York). cDNA synthesis from 2 μg of total RNA was accomplished by extension with dT_18 _in the presence of 200 U M-MLV reverse transcriptase (Invitrogen, Merelbeke, Belgium) for 1 h at 42°C in a final volume of 20 μl. Reaction volume was then adjusted to 100 μl with water. cDNA from HUES human embryonic stem cells at day 5, synthesized as described above, was a kind gift of A. Loriot and C. de Smet (Ludwig Institute for Cancer Research, Brussels).

### Real-time quantitative PCR

Expression levels of β-actin (*ACTB*) [32], *POSTN *and *COL6A3 *were measured by qRT-PCR based on TaqMan technology using the ABI PRISM 7700 Sequence Detection System (Applied Biosystems, Foster City, CA). Primers, probes and qPCR Core Kit reagent were purchased from Eurogentec (Seraing, Belgium). Sequences of primers and probes are described in Table 2. POSTN primers were chosen in exon 3 and 5 respectively, allowing the amplification of all nine *POSTN *splicing variants. Reactions were done in a final volume of 25 μl with 200 nM primers, 100 nM probe, 200 μM dNTPs, 5 mM MgCl_2_, 0.625 U Hot Gold Star polymerase and 2.5 μl cDNA in 1× buffer. Annealing temperature was of 60°C for *POSTN *and *COL6A3 *and of 62°C for *ACTB *[32]. Amplicon sizes were of 186 bp (*POSTN*), 142 bp (*COL6A3*) and 613 bp (*ACTB*). Standard curve equations were established by serial dilutions of PCR-amplified cDNA fragments of *POSTN *(602 bp) and *COL6A3 *(304 bp). cDNA copy numbers were calculated using the following equations: log(cDNA_*ACTB*_) = (Ct-38.5)/3.7; log(cDNA_*POSTN*_) = (Ct-36.9)/3.35 and log(cDNA_*COL*6*A*3_) = (Ct-37.2)/3.4.

**Table 2:** Primers and probes used in qRT-PCR

**Gene**	**Primers**	**Probe (6-FAM/TAMRA)**	**Ref.**
***POSTN***	5'-TGCCCAGCAGTTTTGCCCAT 5'-CGTTGCTCTCCAAACCTCTA	5'-TCCCACGATGCCCAGAGTGCCA	This study
***ACTB***	5'-GGCATCGTGATGGACTCCG 5'-GCTGGAAGGTGGACAGCGA	5'-TCAAGATCATTGCTCCTCCTGAGCGC	[32]
***COL6A3***	5'-GAAGACCGGCAGCTCATCAA 5'-CGATGTTGCAGATGTCCAAGCA	5'-CACAGCAGTGGGGCATGCGCTT	This study

### Immunoblotting analysis

To detect secreted periostin in the supernatants, LB2077-1, LB2730-1 melanoma cells were grown in IMDM medium until 90–100% confluency and thereafter kept in serum-free IMDM for 48 hours. LB2181-2 and LB2667-1a cell lines were used as negative controls. Four ml-supernatants from 400 000 cells were collected after 72 hours and concentrated 10 times using the Microsep centrifugal devices 10 K (Pall Life Sciences, Ann Arbor, MI). Equal volumes of concentrated supernatants were subjected to 7% SDS-PAGE and electroblotted onto a PVDF membrane (Millipore). The membrane was incubated successively with rabbit polyclonal antibody (ab14041, Abcam, Cambridge, UK) specific for periostin and then horseradish peroxidase-conjugated anti-rabbit IgG (Stressgen, Ann Arbor, MI). Protein bands were visualized using the Luminol Reagent (Santa Cruz Biotechnology).

### Statistical analysis

Student *t*-test was applied to compare *POSTN *expression in different sample series.

## List of abbreviations

POSTN, periostin; 

ACTB, β-actin; 

COL6A3, α3 chain of collagen VI; 

qRT-PCR, quantitative reverse transcription-polymerizing chain reaction; 

SCLC, small cell lung cancer; 

NSCLC, non-small cell lung cancer; 

NET, neuroendocrine tumor; 

TGF-β1, transforming growth factor-β1; 

SD, standard deviation; 

PBL, peripheral blood lymphocyte.

## Competing interests

The author(s) declare that they have no competing interests.

## Authors' contributions

GT and MM carried out the quantitative RT-PCR analysis for *POSTN*, *COL6A3 *and *ACTB *genes. MM performed the immunoblot analysis. FB collected tumor samples, established cancer cell lines, synthesised cDNA from a series of samples and helped to draft the manuscript. NVB participated in the melanoma fibroblast marker selection and helped to draft the manuscript. AD conceived the study, participated in its design and coordination, performed the statistical analysis and drafted the manuscript. All authors read and approved the final manuscript.
